# Preparation of mixed-mode stationary phase for separation of peptides and proteins in high performance liquid chromatography

**DOI:** 10.1038/s41598-022-08074-7

**Published:** 2022-03-08

**Authors:** Sarah Alharthi, Ashraf Ali, Muzaffar Iqbal, Aliya Ibrar, Bashir Ahmad, Sobia Nisa, Fazal Mabood

**Affiliations:** 1 Department of Chemistry, College of Science, Taif University, P.O. Box 11099, Taif, 21944 Saudi Arabia; 2 Department of Chemistry, The University of Haripur, Haripur, 22620 KPK Pakistan; 3 Department of Biology, The University of Haripur, Haripur, 22620 KPK Pakistan; 4 Department of Microbiology, The University of Haripur, Haripur, 22620 KPK Pakistan; 5 Institute of Chemical Sciences, University of Swat, Swat, 19200 KPK Pakistan

**Keywords:** Analytical chemistry, Biochemistry, Materials chemistry, Polymer chemistry, Chemical synthesis, Biochemistry, Chemical biology, Chemistry

## Abstract

Porous silica particles were prepared by sol–gel method with some modification to get wide-pore particles. These particles were derivatized with N-phenylmaleimide-methylvinylisocyanate (PMI) and styrene by reversible addition fragmentation chain transfer (RAFT) polymerization to prepare N-phenylmaleimide embedded polystyrene (PMP) stationary phases. Narrow bore stainless steel column (100 × 1.8 mm i.d) was packed by slurry packing method. The chromatographic performance of PMP column was evaluated for the separation of synthetic peptides mixture composed of five peptides (Gly-Tyr, Gly-Leu-Tyr, Gly-Gly-Tyr-Arg, Tyr-Ile-Gly-Ser-Arg, Leucine enkephalin) and tryptic digest of human serum albumin (HAS) respectively. Number of theoretical plates as high as 280,000 plates/m were obtained for peptides mixture at optimum elution condition. Separation performance of the developed column was compared with commercial Ascentis Express RP-Amide column and it was observed that separation performance of PMP column was better than commercial column in terms of separation efficiency and resolution.

## Introduction

In recent years the biopharmaceutical industry has become an expanding global market with a substantially increased market share. The analysis of peptides and proteins is highly desired with the explosive growth of biopharmaceutical industry^1–3^. Several impurities are produced during peptide synthesis in addition to the target peptides, therefore chromatographic purification is required to get peptides of the required purity. Analysis and characterization of proteins in bodily fluids, tissues and cells is a highly challenging task owing to the presence of large number of potentially detectable species in a single sample. Even though mass spectrometry is an effective tool for peptides and proteins sequencing but if such samples are injected at once to mass spectrometer, the separation will be not satisfactory. This issue can be mitigated by the implementation of liquid chromatography (LC) separation prior to MS analysis, which will reduce the number of analytes entering to the mass spectrometer at a given time^4–6^. Furthermore, during the liquid phase separation the analytes can be focused within narrow zones, which concentrate these analytes and increase MS detection sensitivity. Liquid chromatography (LC) has significantly advanced over the past decade and became a prevalent technique in proteomic analysis^7–10^.

Reverse phase liquid chromatography (RP-LC) is widely used for the purification and separation of peptides mixture using octadecyl-modified silica (ODS) as a stationary phase^11–13^. However, RP stationary phases do not provide satisfactory separation of peptides and proteins due to their complex structure and amphoteric nature^14,15^. Therefore, specially designed stationary phases are required for the analysis of peptides and proteins that have both polar and nonpolar moieties to interact with these analytes and retain them^16^. Mixed-mode chromatography which offers multimodal interaction could be an alternative to RP-LC for the separation of peptides, proteins and other complex mixtures. Several mixed-mode stationary phases have been prepared and the columns packed with these stationary phases have been used for peptides and proteins separation^17–21^. Mixed-mode stationary phases (WAX/RPLC, HILIC/RPLC, polar embedded/RPLC) are suitable for peptides and proteins separation owing to the presence of both polar and non-polar groups^22–28^. Similarly, polar-embedded stationary phases with covalently bonded polar groups have shown good separation capability and a unique selectivity for polar and nonpolar analytes, because the separation depends on multimodal interactions between the analyte and the stationary phase^29–32^. Recently, Zhang et al.^30^ have prepared docosyl-terminated polyamine stationary phase and successfully separated hydrocarbons, antidepressants, flavonoids, nucleosides, estrogens and several other analytes. Polar embedded stationary have both polar and non-polar groups therefore it could be used for the separation of peptides and proteins which have both hydrophobic and hydrophilic moieties. Polar embedded columns such as amide embedded C18 columns are commercially available under the trade name Ascentis Express RP-Amide column, but these columns have only been used for the analysis of amines^33^.

In current study, a polar-embedded stationary phase (N-phenylmaleimide embedded polystyrene) was prepared and evaluated for the separation of peptides and tryptic digest of HSA. The following strategies were adopted for the preparation of stationary phase. Porous silica particles were prepared according to the procedure given in our previous publications with some modification in the preparation protocol^31,34–39^.The ratio of urea, polyethylene glycol (PEG), TMOS, water acetic acid was adjusted to prepare silica particles having large pore size. Secondly, a new ligand phenylmaleimide-methylvinyl isocyanate was synthesized and silica particles were derivatized with it to prepare polar-embedded stationary phase. The resultant stationary phase was packed into stainless steel column (100 × 1.8 mm i.d) using an optimized packing protocol. Column packing was assisted with mechanical vibration to ensure the formation of a homogenous bed inside the column. The packed column was evaluated for separation of peptides mixture composed of five peptides; (Gly-Tyr, Gly-Leu-Tyr, Gly-Gly-Tyr-Arg, Tyr-Ile-Gly-Ser-Arg, Leucine Enkephalin) and tryptic digest of Human serum albumin (HAS). It was observed that peptides mixture and tryptic digest of HSA were separated with good resolution and efficiency. The separation performance of PMP column was compared with Ascentis Express RP-Amide column. It was observed that peptides and proteins were separated with good resolution and efficiency on PMP column and the separation efficiency of PMP column was higher than Ascentis Express RP-Amide column.

## Material and methods

### Chemicals and reagents

PEG (polyethylene glycol), urea, acetic acid, trimethoxy orthosilicate (TMOS), Chlorotrimethylsilane(TMCS), trypsin, human serum albumin (HSA), ammonium chloride, urea, hexamethyldisilazane (HMDS), methacryloyl chloride (MC), styrene, 4-hydroxy-TEMPO, benzoyl peroxide (BPO), HPLC grade acetonitrile (ACN), methanol, 2-propanol, and acetone were purchased from Sigma–Aldrich (St. Louis, MO, USA).

### Synthesis of porous silica particles

A mixture of urea (8 g), polyethylene glycol (8 g) and 8 mL 0.01 N acetic acid was stirred for 10 min and 24 mL TMOS was added into it under ice-cold condition. The reaction mixture was heated at 40 °C for 6 h followed by heating at 120 °C for 8 h in a stainless steel autoclave. Water was decanted and the residual material was dried at 70 °C for 12 h. The dried soft bulk was smoothly grounded and calcined at 550 °C for 12 h in a furnace. Three batches were prepared and characterized to check the reproducibility in terms of particle size, pore size and surface area of the particles.

### Synthesis of polar-embedded RP stationary phase

A stationary phase containing both polar groups and polystyrene chain was prepared by the surface modification of silica particles with a pre-synthesized ligand phenylmaleimide-methylvinylisocyanate (PCMP) followed by radial polymerization with styrene. The preparation procedure is described below.

#### Preparation of phenylmaleimide-methylvinylisocyanate (PMCP)

Phenylmaleimide-methylvinylisocyanate copolymer (PMCP) was prepared by dissolving N-phenylmaleimide (200 mg) and methylvinylisocyanate (100 mg) in anhydrous toluene, then 0.1 mL of 2,2'-azoisobutyronitrile (AIBN) were added into the reaction flask through dropping funnel. The mixture was heated at 60 °C for 3 h, filtered and dried at 40 °C in oven for 3 h.

#### Functionalization of silica particles with PMCP

Dried silica particles (2 g) were dispersed in dry toluene (100 mL), stirred and sonicated for 10 min in 500 mL round bottom flask. PMCP (10 mg) was dissolved in toluene and added drop-wise into the reaction flask through a dropping funnel. The mixture was refluxed at 100 °C for 8 h, filtered and washed with acetone and dried at 60 °C for 3 h. Then, PMCP bonded silica particles (100 g) was dissolved in toluene (200 ml), then 4-hydroxy-TEMPO (2 mL) was added into it in the presence of 100 µL dibutyl-tin-dilaurate as a catalyst. The mixture was stirred at 50 °C for 8 h, filtered and dried at 50 °C for 3 h.

#### Radical Polymerization of Styrene

**Figure 1:**
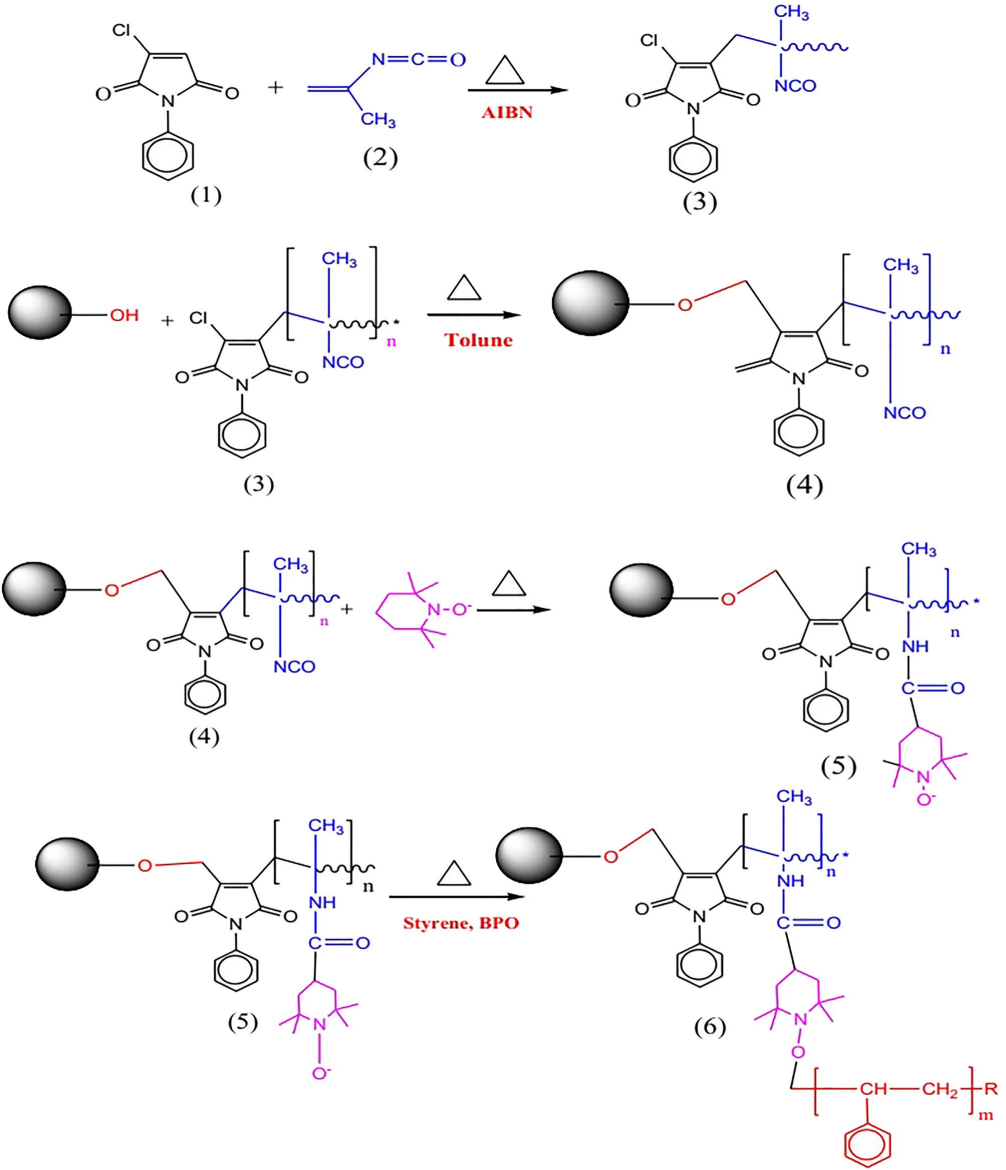
Reaction scheme for the preparation of mixed-mode stationary phase.

Styrene (1 mL), benzoyl peroxide BPO (0.5 mL) and TEMPO-PMCP attached silica particles (1.5 g) were dispersed in toluene and purged with nitrogen. The polymerization of styrene was carried out at 100 °C for 12 h. The resultant product was washed with methanol and dried at 60 °C overnight. The overall reaction scheme is given in the Fig. 1.

### Characterization of the stationary phase

The samples were degassed at 393 K for 1 h to obtain a residual pressure of less than 10^−3^ Torr. The amount of N_2_ adsorbed at a relative pressure of P/P0 = 0.99 was used to determine the total pore volume. The morphology of bare and ligand bonded silica particles was examined with scanning electron microscope (Hitachi High Technologies, Tokyo, Japan). The dried samples (bare silica and ligand bonded silica particles) were put on aluminum stubs using adhesive carbon tape. A Q150T sputter coater was used to coat gold over the sample and 5 nm Au layer was deposited over the sample. This enhances the efficiency of the process using low voltages and giving a fine-grain, cool sputtering. A Thermo Electron (Waltham, MA, USA) Flash EA1112 elemental analyzer was used to carry out elemental analysis. A Malvern (Worcestershire, UK) Mastersizer 2000 particle size analyzer was used to get particle size distribution. Bare silica particles and ligand bonded silica particles (5 mg each) were dispersed in 5 mL isopropyl alcohol, sonicate for 10 min, vortexes for 5 min, and subjected to the optical bench of Mastersizer. Thermo gravimetric analysis were carried out over a temperature range of 30 to 800 °C at 5 °C per minute.

### Column packing

Glass-lined stainless-steel narrow-bore column of dimensions (100 × 1.8 mm i.d.) was packed using slurry packing method, the same procedure was applied as used in Ref.^31^. A stainless steel column (glass lined, 100 × 1.8 mm i.d.) with an outlet union containing 1 µm screen frit was connected to the slurry packer (Alltech Deerfield, IL, USA). The slurry of stationary phase was prepared by suspending the 150 mg stationary phase in 1.2 mL methanol, and fed into the reservoir column. Methanol was used as slurry solvent as well as pushing solvent. The columns were packed by applying a pressure sequence of 100 MP for 10 min, 80 MP for 15 min, and 60 MP for 30 min. Mechanical vibration was applied with two GC column vibrators (Alltech, Deerfield, IL, USA) during packing to ensure the homogenous packing of column. Slurry packer was closed and the pressure was released slowly to prevent any damage inside the column. Column was disconnected from the slurry packing set-up and another union was connected to the inlet and connected to LC system to check its performance.

### HPLC analysis

A special µLC set-up was constructed using LC pump (10AD Shimadzu, Japan), an injector with 50nL injection loop (Valco (USA) C14 W.05), membrane degasser (Shimadzu DGU-14A), a UV–VIS capillary window detector (UV-2075), and glass lined micro-column. Very narrow and short connecting tubing were used to minimize the effect of extra column band broadening. After packing, the 1/16" outlet of the reducing union was installed with a capillary (50 μm i.d. 365 ith a capillary (50 μm) of the reducing union was installed. Data collection and chromatogram processing were accomplished with Multichro 2000 software. The UV absorbance of the test analytes was monitored at 254 nm. The chromatographic data was analyzed by OriginPro8 (Northampton, MA).

### Preparation of tryptic digest of Human Serum Albumin (HSA)

Albumin from human serum, lyophilized powder, ≥ 96% (agarose gel electrophoresis) 3 mg was mixed with trypsin (1.5 mg), 4.0 M urea (1 mL), and 0.2 M ammonium bicarbonate (1 mL). The solution was stirred for 10 min and kept in water bath at 37 °C for 6 h followed by quenching with 1 mL 0.1% TFA. The solution was filtered and stored below 4 °C.

### Columns evaluation

PMP column was evaluated for the separation of peptides mixture and tryptic digest of HSA respectively. PMP column was checked for the separation of peptides mixture and tryptic digest of HSA and the results were compared with Ascentis Express RP-Amide column. Numbers of theoretical plates were calculated by the following equation:

1 $$N=5.54 ({\frac{tr}{w ( \frac{1}{2})} )}^{2}$$

where w_1/2_ is the bandwidth at half height and tr is the retention time.

## Results and discussion

### Morphology of the stationary phase

**Figure 2:**
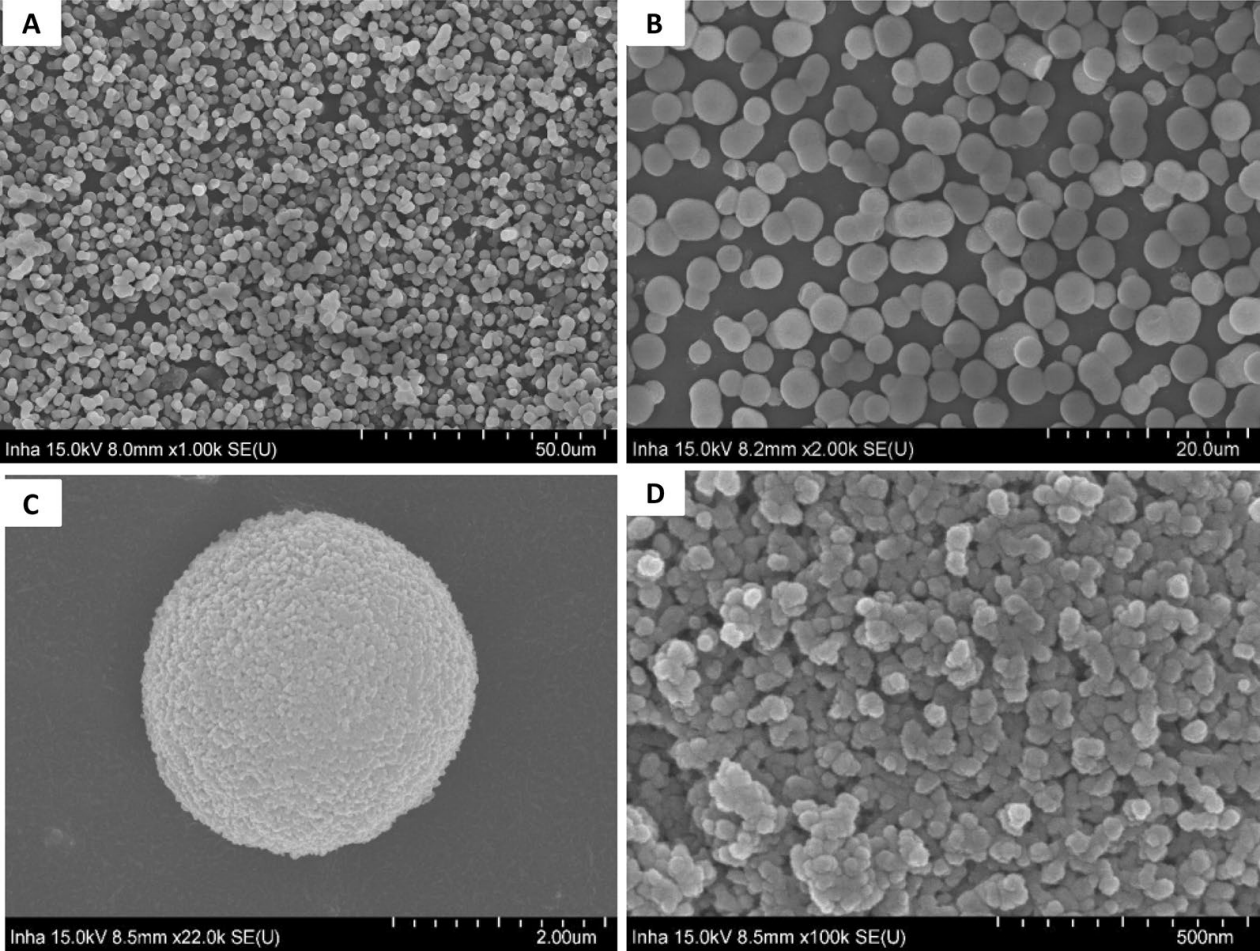
Scanning electron microscopic images of bare silica particles (**A**, **B**) and ligand bonded silica particles (**C**, **D**).

The SEM images of bare silica particles and ligand bonded silica particles are shown in Fig. 2. SEM images of bare silica particles (A,B) show that these particles are spherical in shape as compared to our previous study where the particles were elongated or having an irregular symmetry. The surface of ligand bonded silica particles (C, D) are smoother than bare silica particles which may be owing to the coating of polystyrene chain over the surface of silica particles.

### Particle size distribution

**Figure 3:**
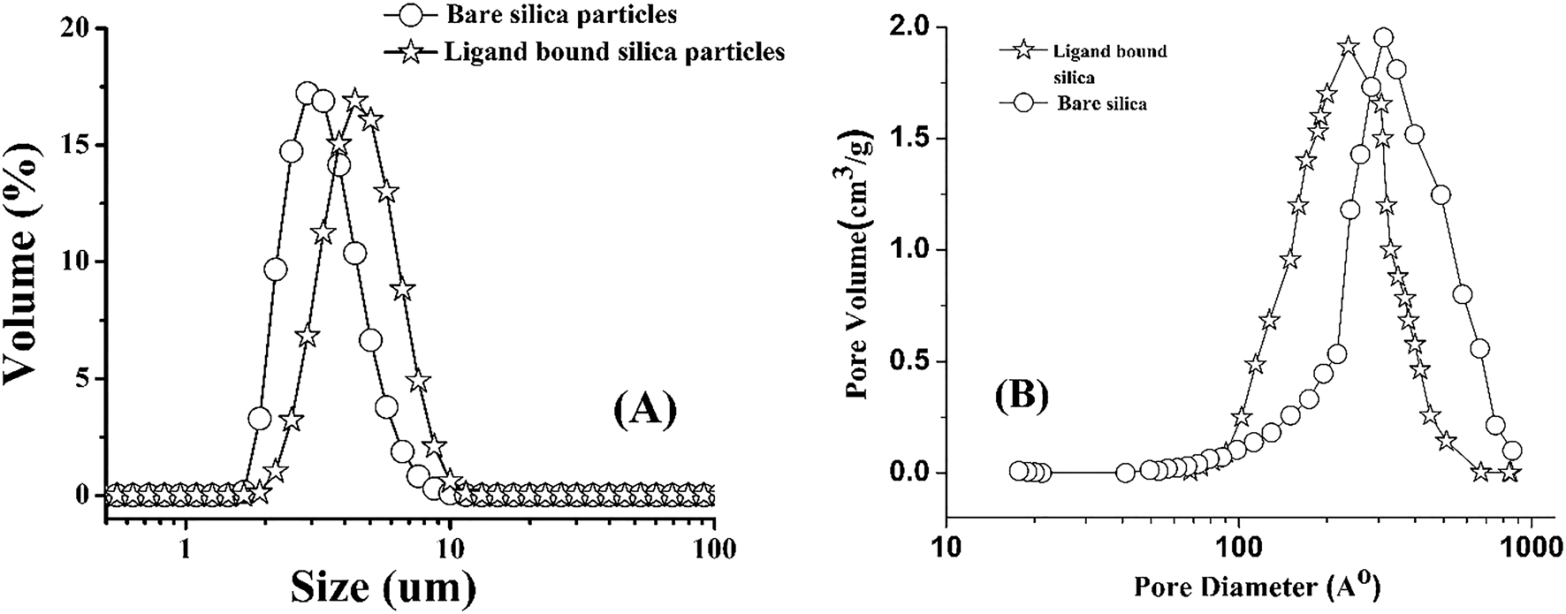
Particle size distribution (**A**) and pore size distribution (**B**) of bare silica particles and ligand bound silica particles.

The particle size distribution of bare silica particles and ligand bonded silica particles is shown in Fig. 3(A). The volume-based particle size distribution curves show that the size of silica particles increases after chemical modification (Fig. 3A). The particles size distribution data of silica particles of the current study and previous study are compared in Table 1(A). The volume-based particle size d(0.5) of PMP is 3.36 μm while d(0.5) value of our previous study was 3.05 μm (polystyrene bound silica particles)^34^. The particle size distribution of this batch is narrow as compared to our previous study^34^ due to change in the ratio of PEG, urea, TMOS and acetic acid in reaction mixture. The particles size of PMP phase is little bigger than polystyrene bound silica particles phase of our previous study. It means that the surface functionalization of silica particles with styrene only have deposited a polystyrene layer (0.97 µm) over the silica surface while in PMP phase the layer thickness is 1.38 µm.

**Table 1:** Particle size distribution (A) and pore size distribution (B) of bare ligands bound silica particles.

(A) Particle size distribution									
	Bare silica particles			Ligand bound silica particles (Current study)			ligand bound silica particles (Previous study)^34^		
Particle size (Volume based)	d (0.1)	d (0.5)	d(0.9)	d (0.1)	d (0.5)	d(0.9)	d (0.1)	d(0.5)	d(0.9)
	0.96	1.46	3.01	1.65	3.36	6.27	1.56	3.05	7.62

(B) Pore size distribution									
Pore size	Bare Silica particles				Ligand bound silica particles				
	Previous studies			Current study	Previous studies			Current study	
	^40^	^41^	^34^		^40^	^41^	^34^		
Pore size (Å)	363	296	295	310	303	216	232	241	
Pore volume (cm^3^/g)	1.09	0.75	0.73	0.67	0.95	0.63	0.61	0.58	
Surface area (m^2^/g)	110	126	124	116	83	115	111	105	

### Pore size distribution of bare and ligand bound silica particles

The pore size, pore volume and surface area of silica particles of the current this study are given in Table 1(B). The PSD plots of bare silica particles and ligand bonded silica particles are shown in Fig. 3(B). The results are comparable to our previous study^34^. The pore size of bare and ligand bound silica particles is 310 Å and 241 Å respectively, which indicated that after chemical modification the pore size decreased by 69 Å as shown in Table 1(B) and the curve’s shift can be seen in Fig. 3(B). Similarly, the pore volume of silica particles decreased from 0.67 to 0.58 cm^3^/g after chemical modification. The specific surface area of silica particles of current study is 116 m^2^/g which is comparable to that of our previous study (124 m^2^/g). The surface area (m^2^/g) of silica particles also decreased from 116 m^2^/g to 105 m^2^/g after chemical modification as shown in Table 1(B).

### Characterization of PMP stationary phase

**Figure 4:**
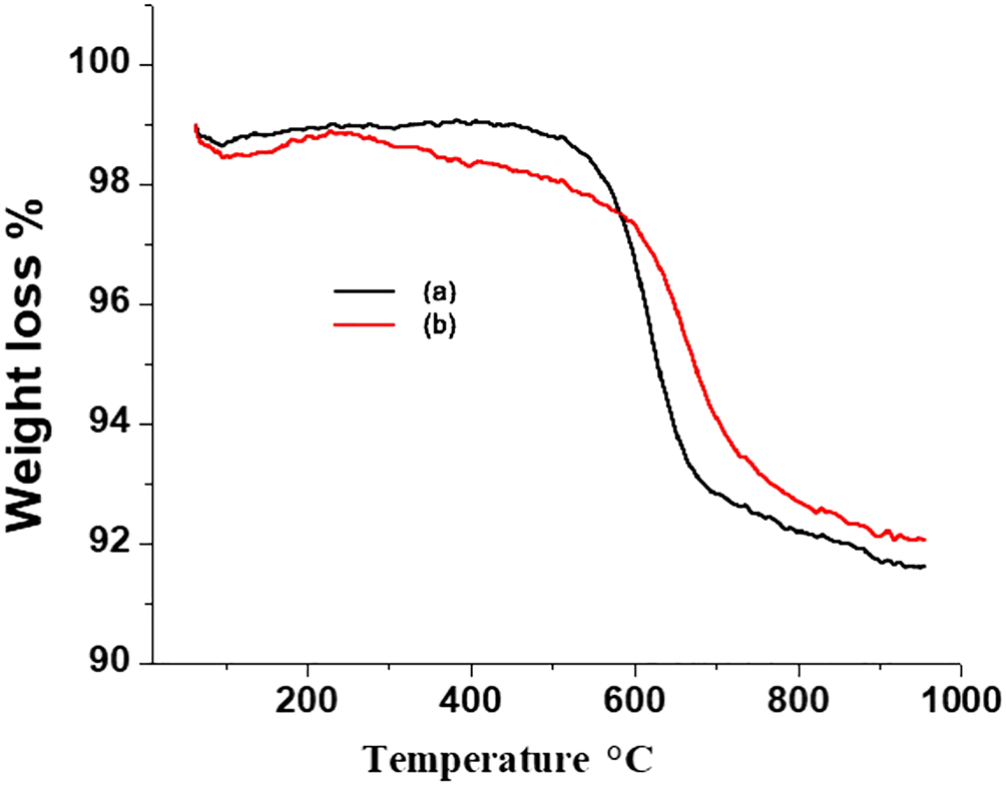
TGA of PMP stationary phase, product 5 (**a**), product 6 (**b**).

The elemental analysis results of the stationary phase is shown in Table 2. The carbon load of current stationary phase is 6.35%, which is less than those of our previous studies, (polystyrene bonded silica particles, which were 7.93%^35^ and 10.21 %)^42^. The carbon load of the current stationary phase is low because some polar ligands such as phenylmaleimide-methylvinylisocyanate (PCMP) and 4-hydroxy-TEMPO were used in addition to styrene in the preparation of current SP. The nitrogen weight % of current stationary phase is 2.21% while that of previous studies were 0.17^35^ and 0.85%^42^ respectively. It means that the wt % of nitrogen is high in the current stationary phase owing to phenylmaleimide. Similarly the carbon loads of product (4) and (5) are 2.7% and 2.9% respectively, while the carbon load of final product (6) is 6.35% as shown in Table 2. Thermo gravimetric analysis (TGA) was carried out for PMP stationary phase to check the weight loss, and the TGA curve is shown in Fig. 4. The TGA curve shows the weight loss of 8.6% which is in good agreement with the carbon load (6.35%) since the ligand contains not only C but also N, O, and H.

**Table 2:** Elemental analysis of polystyrene bound silica particles.

Element	Weight %				
	Current study ^a^	Current study ^b^	Current study ^c^	Ref.^35^	Ref.^42^
Carbon	2.7%	2.9%	6.35%,	7.93%	10.21%
Nitrogen	1.11	1.32	2.21%	0.17	0.85
Sulfur	–	–	–	0.84	2.12

^a^Corresponds to the product (4) in Fig. 1 (reaction scheme).

^b^Corresponds to the product (5) in Fig. 1 (reaction scheme).

^c^Corresponds to the product (6) in Fig. 1 (reaction scheme).

Phenylmaleimide-methylvinylisocyanate ligand was chosen for surface modification of silica particles because it have a polar phenylmaleimide group and vinyl isocyanate group. Vinyl isocyanate group can be further reacted with styrene via living radical polymerization. The second reason was to insert a group which have moderate interaction with analyte and do not have strong electrostatic interactions between analytes and stationary phase since the phenylmaleimide moiety have no virtual charge at normal pH. The polarity of the stationary phase can be controlled by optimum amount of styrene and reaction time of radical polymerization. The last step of reaction (radical polymerization) is very critical which can change the polarity of stationary phase. Elemental analysis were carried out to check the carbon load of these stationary phases. It was observed that increasing the amount of styrene and reaction time increased the carbon load of stationary phase and vice versa. SP prepared with different concentration of styrene have different carbon loads. Similarly these stationary phases were packed in stainless steel column and their chromatographic performance (selectivity, resolution, N values etc.) were checked. Based on these experiments an optimized formulation was selected for preparation of PMP stationary phase to secure both controlled polarity and good analyte retention.

### Chromatographic performance of PMP columns

#### Separation of synthetic peptides

**Figure 5:**
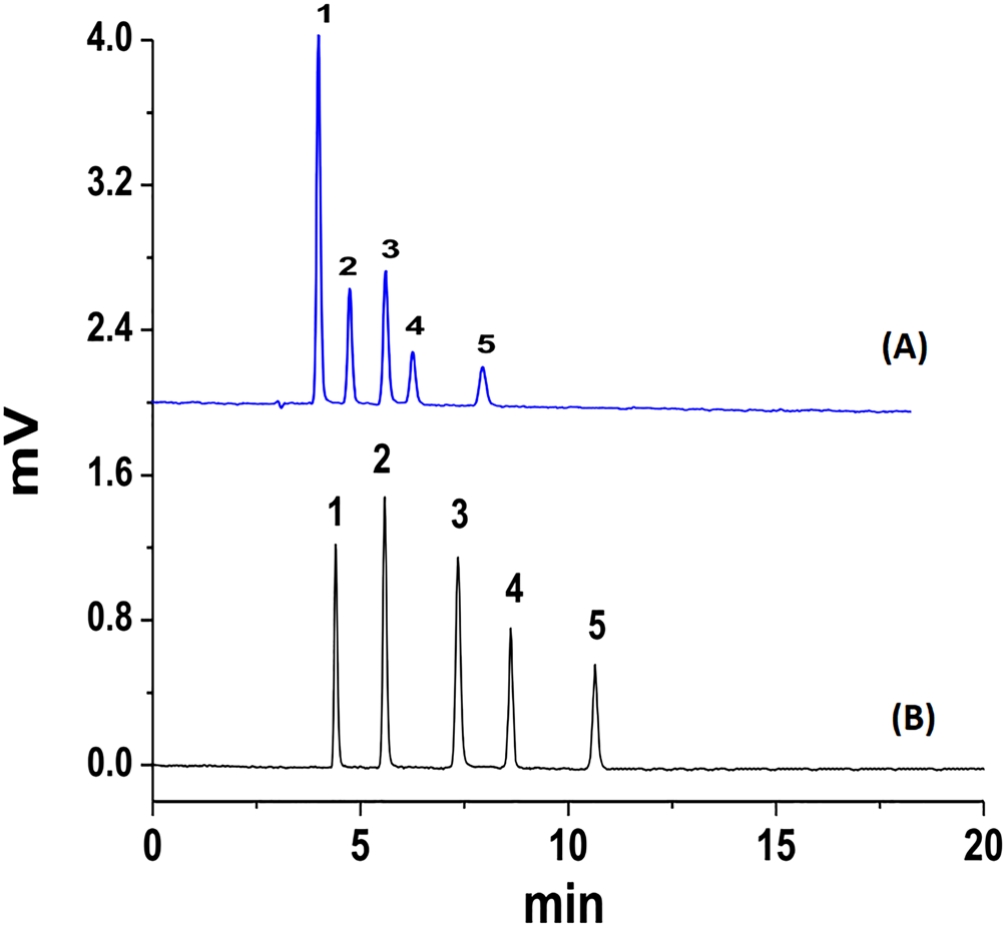
Separation of peptides mixture on PMP Column (**B**) and Ascentis Express RP-Amide column (**A**); mobile phase 60/40 ACN/ H_2_O (TFA 0.1%), PMP column dimension (100 × 1.8 mm i.d); The elution order of analytes: 1(Gly-Tyr), 2 (Gly-Leu-Tyr), 3(Gly-Gly-Tyr-Arg), 4 (Tyr-Ile-Gly-Ser-Arg) and 5 (Leucine Enkephalin).

The PMP column was also evaluated for the analysis of five peptides mixture (Gly-Tyr, Gly-Leu-Tyr, Gly-Gly-Tyr-Arg, Tyr-Ile-Gly-Ser-Arg, Leucine Enkephalin) using a mobile phase; 60/40 (v/v) ACN/water (0.1% TFA) at the flow rate of 80 μL/min. Numbers of theoretical plates (N) 20,000 ± 100 per column (100 × 1.8 mm i.d.) were obtained (200,000 plates/m) at optimum elution conditions. The N values of three PMP columns are given in Table 3 and the chromatograms are shown in Fig. 5A. Fast analysis was carried out at high flow rate (700 μL/min) on PMP column and five peptides were eluted within one minute with quite good N value 13,500 ± 330 per column (100 × 1.8 mm i.d.) corresponding to 135,000 plates/m (Fig. 5B). Three columns of the same dimensions (100 × 1.8 mm i.d. were packed from three different batches of PMP stationary phases to check the reproducibility. The same test mixture was separated on each column using optimum elution conditions and the number of theoretical plates N and retention times of analytes were recorded for each column. The reproducibility data of PMP columns are shown in Table 4. There is a good correlation in reproducibility of PMP columns with very low %RSD values as shown in Table 3.

**Table 3:** Comparison of number of theoretical plates (N) for PMP column and Ascentis Express RP-Amide column at optimum flow rate, column dimensions (100 × 1.8 mm i.d) and elution condition: ACN: H_2_O 60:40 (0.1% TFA), flow rate (80 µL/min).

Peptide	PMP Column current study	Ascentis Express RP-Amide column
Gly-Tyr	30,600 ± 100	19,500 ± 400
Gly-Leu-Tyr	30,100 ± 200	19,300 ± 500
Gly-Gly-Tyr-Arg	29,100 ± 250	18,300 ± 300
Tyr-Ile-Gly-Ser-Arg	29,000 ± 300	15,200 ± 200
Leucine Enkephalin	28,100 ± 200	15,000 ± 100

**Table 4:** Column to column & day to day reproducibility, N values of three PMP columns at optimum flow rate, column dimensions (150 × 1.8 mm i.d.) and elution condition: ACN: H_2_O 60:40 (0.1% TFA), flow rate (80 µL/min).

Analyte	Reproducibility (Batch to batch)		Reproducibility (per days)	
	N values	%RSD	N values	%RSD
Gly-Tyr	30,900 ± 400	0.08	30,700 ± 100	0.10
Gly-Leu-Tyr	30,800 ± 300	0.14	30,300 ± 200	0.13
Gly-Gly-Tyr-Arg	30,100 ± 700	0.16	30,000 ± 400	0.16
Tyr-Ile-Gly-Ser-Arg	29,500 ± 400	0.26	29,300 ± 100	0.20
Leucine Enkephalin	29,200 ± 300	0.33	29,000 ± 200	0.24

#### Separation of tryptic digest of Human Serum Albumin (HSA)

**Figure 6:**
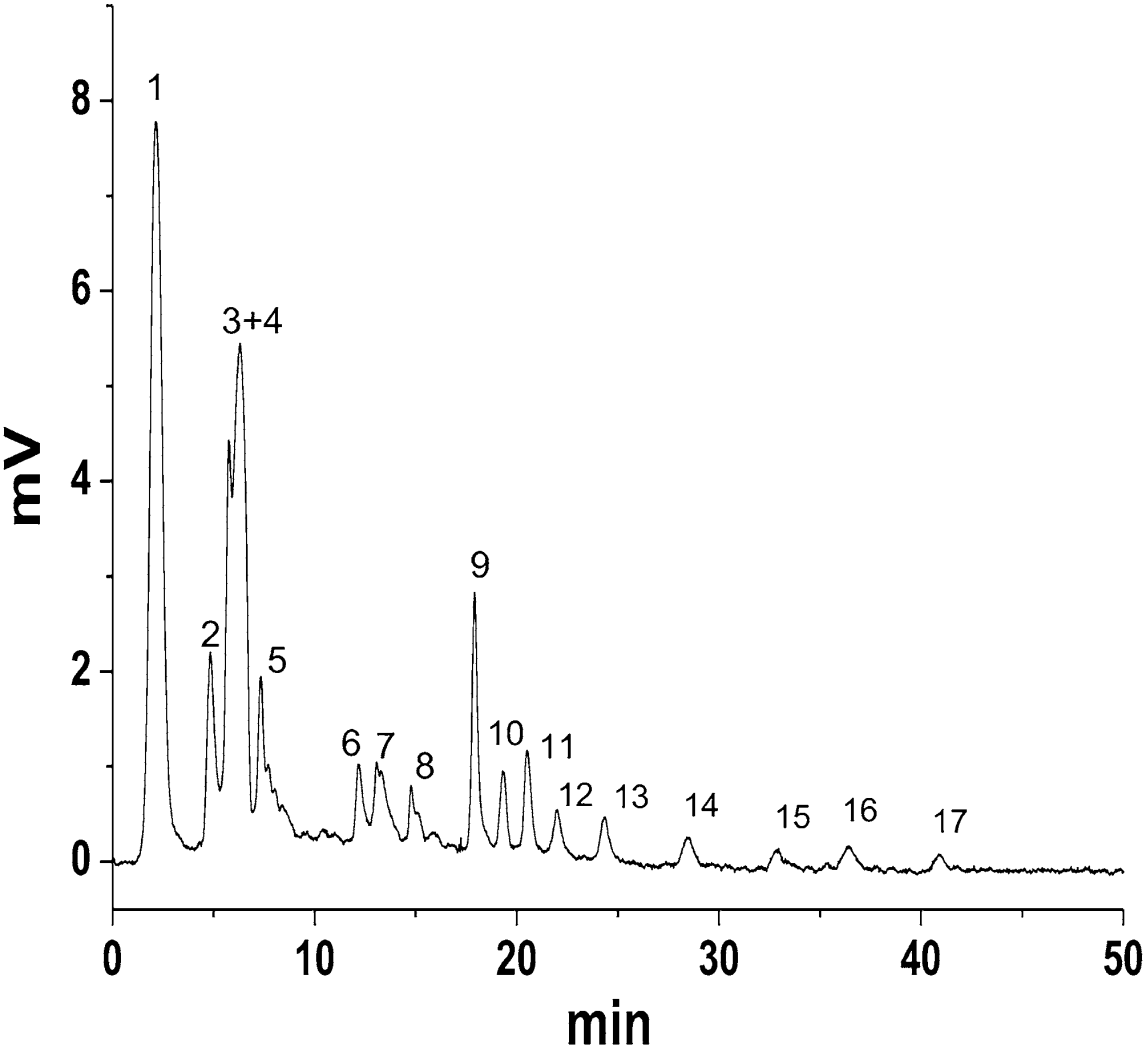
Separation of tryptic digest of HSA on PMP column (100 × 1.8 mm i.d); flow rate (100 µL/min), mobile phase 60/40 acetonitrile/ water with 0.1% TFA.

PMP column (100 × 1.8 mm i.d) was evaluated for the separation of tryptic digest of human serum albumin in high performance liquid chromatography. The chromatogram in Fig. 6 shows that the sample is separated well with quite good resolution. The HSA digest was analyzed using flow rate of 100 µL/min, mobile phase 70/30 acetonitrile/water with 0.1% TFA. The HSA digest has been separated into 17 peaks as shown in the chromatogram **(**Fig. 6) which corresponds to 17 peptides. The separation efficiency of individual peaks from HSA digest are calculated and the values are given in Table 5.

**Table 5:** Retention time (Rt) and number of theoretical plates (N) values of peptides in tryptic digest of human serum albumin (HAS). Column: SMP column, column dimensions (50 × 1.8 mm i.d) and elution condition: ACN: H_2_O 60:40 (0.1% TFA), flow rate (80 µL/min).

Peak number	Retention time	N value
1	3.2	22,700
2	5.3	25,300
3	7	15,300
4	7.5	20,100
5	12.5	18,300
6	18	24,700

### Permeability of PMP column

Column permeability was calculated using the following equation.

2 $$\mathrm{K}=\frac{\mathrm{u} \eta \mathrm{L} }{\Delta\mathrm{P}}$$

where L is the column length, η is the viscosity of the mobile phase, ΔP is the column back pressure and *u* is mobile phase linear velocity. The permeability of PMP column was 2.5 × 10^−14^ m^2^ at 25 μL/min using 60/40 v/v ACN/water. The permeability of the PMP column (100 × 1.8 mm i.d) is similar to that of our previous study Ref.^34^. The permeability of the columns packed with superficially porous particles were noted to be 1.7 × 10^−15^ for 1.3 μm particles, 3.1 × 10^−15^ for 1.7 μm particles, 5.2 × 10^−15^ for 2.6 μm particles and 2.5 × 10^−14^ m^2^ for 5 μm particles^43^. So, the permeability PMP phase is similar to the permeability of 5 μm core–shell particles.

### Porosity of PMP column

Porosity was calculated by the following equation using methanol and chloroform as filling solvents

3 $$V0=\frac{Wx - Wy }{\rho{\mathrm{x}}-\rho{\mathrm{y}}}$$

where *Wx* is the weight of the column filled with chloroform and Wy is the weight of column filled with methanol, ρ is the density of the solvent. Density of methanol (ρ = 0.7866) and that of chloroform (ρ = 1.484). The total porosity of SILICA PARTICLES -C18 column (100 × 1.8 mm i.d)^34^ and C18-Urea column^31^ of our previous study were found to be 0.63 and 0.55 respectively. It means that the presence of urea ligand has decreased the permeability of stationary phase. On the other hand the total porosity of PMP column (100 × 1.8 mm i.d) was 0.60. The permeability PMP column is less than C18 bonded silica particles packed column because in C18 types stationary phases the C18 ligands attached to silica particles in the form of linear chains while in polystyrene types stationary phases relatively thick layer of polymer form around silica particles. In a typical experiment the column porosity was calculated as,

4 $$Porosity = \frac{Void\,volume}{Colum\,volume}= 0.63$$

### Van Deemter plots

**Figure 7:**
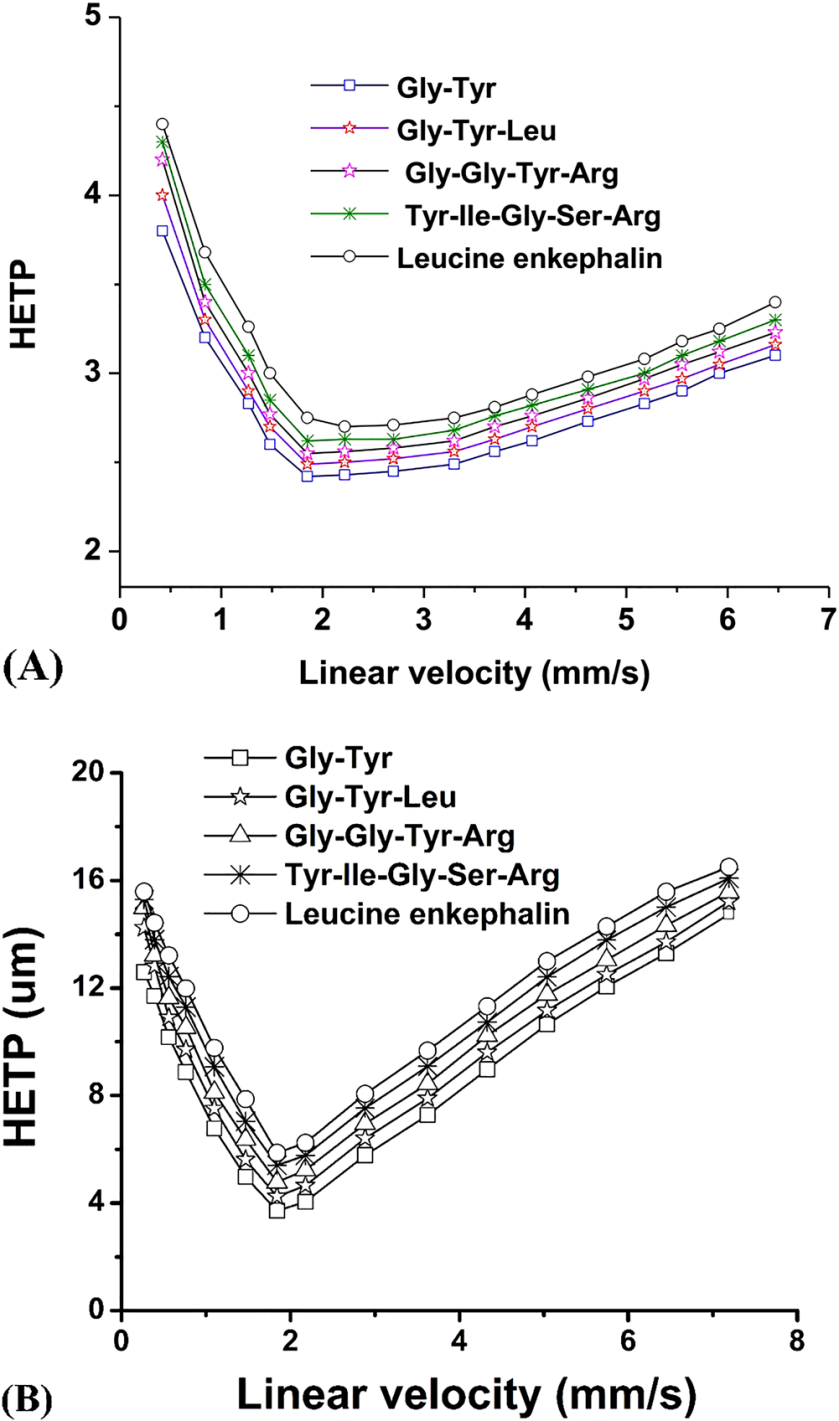
(**A**) The van Deemter plots (HETP vs mobile phase linear velocity) obtained with PMP column (100 × 1.8 mm i.d) in 60/40 ACN/H_2_O with 0.1% TFA. (**B**) The van Deemter plots (HETP vs mobile phase linear velocity) obtained with Ascentis Express RP-Amide column (100 × 1.8 mm i.d) in 60/40 ACN/H_2_O with 0.1% TFA.

Figure 7A, B shows the van Deemter plot of PMP column (100 × 1.8 mm i.d) and Ascentis Express RP-Amide column (100 × 1.8 mm i.d) respectively using the same elution conditions i.e. 60/40 ACN/H_2_O with 0.1% TFA. Separation of the selected peptides mixture (Gly-Tyr, Gly-Leu-Tyr, Gly-Gly-Tyr-Arg, Tyr-Ile-Gly-Ser-Arg, Leucine Enkephalin) was carried out at various flow rates ranging from 20 µL/min to 800 µL/min on both columns. The minimum HETP values at optimum flow rate (80 µL/min) for PMP column and Ascentis Express RP-Amide column were found to be 2.6 µm and 3.9 µm respectively. The HETP values shows that the separation efficiency of PMP column (100 × 1.8 mm i.d) is much better than commercially available Ascentis Express RP-Amide column (100 × 1.8 mm i.d). The van Deemter plot in Fig. 7(A) shows that the decrease in N value with increase in flow rate is not significantly high as compared to our previous study^34^. Better separation efficiency of the PMP column (100 × 1.8 mm i.d) in comparison to Ascentis Express RP-Amide column is based on the improvement in particles shape, size and sophisticated column packing procedure used in the current work^34^.

## Conclusions

A polar embedded polystyrene stationary phases was prepared and evaluated for the separation of synthetic peptides mixture and tryptic digest of human serum albumin (HAS) in high performance liquid chromatography. The chromatographic performance of PMP column for peptides mixture is excellent in terms of separation efficiency and resolution. The enhanced separation performance of PMP column is owing to several reasons such as particle size and pore size of silica particles, controlled synthesis of the stationary phase and sophisticated column packing. In addition to high separation efficiency, column back pressure was low at high flow rates, which is an additional advantage of this stationary phase. The PMP column exhibited good repeatability and could be applied for the analysis of peptides mixture and tryptic digest of various proteins. We intend to apply this column for the separation of natural products, bioactive compounds from medicinal plants and fungi extracts in liquid chromatography. In future the PMP column will also be evaluated for the separation of proteins and monoclonal antibodies.

## Abbreviations

PMP: N-phenylmaleimide embedded polystyrene
RP: Reversed phase
HILIC: Hydrophilic interaction liquid chromatography
ODS: Octadecyl-modified silica
RAFT: Reversible addition fragmentation chain transfer polymerization
SI-ATRP: Surface initiated-atom transfer radical polymerization
DBTD: Dibutyltin dichloride
HAS: Human serum albuim
PMCP: Phenylmaleimide-methylvinylisocyanate copolymer
